# OnabotulinumtoxinA Improves Sleep Quality in Patients with Chronic Migraine: A Prospective Real-World Study

**DOI:** 10.3390/toxins18090379

**Published:** 2026-09-03

**Authors:** Giovanna Viticchi, Lorenzo Falsetti, Marco Bartolini, Sergio Salvemini, Gioacchino De Vanna, Eleonora Beccacece, Najdi Tanuzi, Mauro Silvestrini

**Affiliations:** 1 Neurological Clinic, Marche Polytechnic University, Via Conca 71, 60126 Ancona, Italy; g.viticchi@staff.univpm.it (G.V.); m.bartolini@staff.univpm.it (M.B.); sergio.salvemini@ospedaliriuniti.marche.it (S.S.); gioacchino.devanna@ospedaliriuniti.marche.it (G.D.V.); eleonora.beccacece97@gmail.com (E.B.); najditanuzi@gmail.com (N.T.); 2 Department of Internal Medicine, Marche Polytechnic University, Via Conca 71, 60020 Ancona, Italy; l.falsetti@staff.univpm.it

**Keywords:** onabotulinumtoxinA, migraine chronification, sleep quality, Pittsburgh Sleep Quality Index, calcitonin gene-related peptide, central sensitization, migraine prevention, sleep disturbances

## Abstract

Sleep disturbances are highly prevalent in patients with chronic migraine (CM) and are increasingly recognized as major contributors to disease burden and headache chronification. Growing evidence suggests that dysregulation of calcitonin gene-related peptide (CGRP) signaling and central sensitization may represent common biological mechanisms linking migraine and sleep impairment. OnabotulinumtoxinA (OBT-A), an established preventive treatment for CM, inhibits the release of CGRP and other nociceptive mediators from trigeminal sensory neurons, thereby reducing peripheral and central sensitization. However, its effects on sleep quality remain incompletely characterized. The aim of this prospective real-world observational pilot study was to evaluate the impact of OBT-A treatment on sleep quality in patients with CM. Consecutive patients initiating OBT-A therapy at a tertiary headache center were enrolled and assessed at baseline (before the first OBT-A treatment cycle) and after three months, immediately before the second scheduled administration, using the Pittsburgh Sleep Quality Index (PSQI), alongside measures of migraine frequency, acute medication use, migraine-related disability, and headache impact. Fifteen patients completed follow-up evaluations. OBT-A treatment was associated with significant improvements across all clinical outcomes. Median monthly migraine days decreased from 18 to 10 (adjusted *p* = 0.006), while acute medication use, MIDAS scores, and HIT-6 scores were significantly reduced. Sleep quality improved markedly, with median PSQI scores decreasing from 10 to 6 (adjusted *p* = 0.006). Eleven of 15 patients (73.3%) achieved a ≥3-point reduction in PSQI, and the proportion of participants with pathological sleep quality (PSQI > 5) decreased from 86.7% to 53.3%. Although this categorical improvement did not reach statistical significance (exact *p* = 0.063), likely due to the limited sample size, it may nonetheless suggest a beneficial effect on sleep quality, although the lack of statistical significance warrants cautious interpretation. These findings provide preliminary real-world evidence that OBT-A treatment is associated with improved subjective sleep quality in patients with chronic migraine. Improvements in sleep quality occurred alongside reductions in migraine frequency, acute medication use, disability, and headache impact. Given the observational and uncontrolled design of the study, it remains unclear whether sleep improvement reflects a direct effect of OBT-A or occurs secondarily to improvements in migraine-related outcomes. Larger prospective controlled studies are needed to clarify the mechanisms underlying sleep improvement and its relationship with treatment response.

## 1. Introduction

Chronic migraine (CM) is a highly disabling neurological disorder characterized by frequent headache attacks and a substantial burden of comorbid conditions. Among these, sleep disturbances are particularly prevalent and clinically relevant, contributing to increased disability, impaired quality of life, and enhanced central sensitization. The relationship between migraine and sleep appears to be bidirectional: poor sleep quality may facilitate migraine chronification, while recurrent migraine attacks can further disrupt sleep architecture and circadian regulation [1]. Increasing evidence suggests that this interaction is supported by shared neurobiological mechanisms involving hypothalamic dysfunction, alterations of circadian networks, dysregulation of sleep–wake regulatory systems, and altered neuropeptide signaling, particularly involving the calcitonin gene-related peptide (CGRP) pathway [2,3,4].

CGRP, a key mediator of migraine pathophysiology, has also emerged as a potential modulator of sleep–wake regulation. Beyond its well-established role in nociceptive transmission and trigeminovascular activation, CGRP is expressed within hypothalamic and brainstem circuits involved in arousal, sleep homeostasis, and circadian rhythms. Accordingly, recent studies have suggested that therapies targeting the CGRP pathway may exert beneficial effects on sleep quality, although the underlying mechanisms remain incompletely understood and evidence remains limited [3]. OnabotulinumtoxinA (OBT-A) is an established preventive treatment for CM and has demonstrated sustained efficacy in reducing headache frequency, migraine-related disability, and overall disease burden. Current European Headache Federation guidelines support the use of OBT-A as an effective and well-tolerated preventive treatment for chronic migraine and provide practical recommendations for patient selection, administration, and treatment monitoring [5]. Its therapeutic action is primarily mediated through the cleavage of SNAP-25, a component of the soluble N-ethylmaleimide-sensitive fusion protein attachment protein receptor complex, resulting in the inhibition of vesicular release of neurotransmitters and neuropeptides involved in migraine pathophysiology, including CGRP, glutamate, and substance P. By attenuating peripheral nociceptive signaling, OBT-A may reduce trigeminovascular activation and limit the development of central sensitization, a hallmark of migraine chronification that is increasingly recognized as a key mechanism linking migraine persistence, pain amplification, and several disease-related comorbidities [6,7,8,9,10,11].

Importantly, growing evidence suggests that OBT-A and CGRP-targeted therapies may converge on partially overlapping biological mechanisms within trigeminal sensory pathways. Experimental studies have demonstrated that OBT-A inhibits the activity-dependent release of CGRP from trigeminal sensory neurons and peripheral nerve terminals, thereby attenuating neurogenic inflammation and peripheral sensitization [6,7]. This functional interaction with the CGRP signaling cascade provides a biologically plausible framework for understanding not only the preventive efficacy of OBT-A in migraine but also its potential influence on migraine-associated symptoms and comorbidities, including sleep disturbances. Emerging evidence further indicates that OBT-A may indirectly modulate central pain-processing networks and neuroinflammatory pathways, contributing to a reduction in central sensitization and potentially influencing sleep-related outcomes [7,8,9,10,11]. Despite the biological rationale linking CGRP signaling, central sensitization, and sleep regulation, the potential impact of OBT-A on sleep disturbances in CM remains insufficiently explored. Available studies are limited by heterogeneous methodologies and by the inconsistent use of validated sleep assessment tools. Consequently, prospective real-world data evaluating changes in sleep quality during OBT-A treatment are scarce, and the extent to which sleep improvement contributes to the overall therapeutic response remains unclear. Addressing this knowledge gap is clinically important because sleep quality may represent both an additional dimension of treatment benefit and a potential marker of therapeutic effectiveness. Therefore, the aim of the present prospective real-world observational pilot study was to investigate the effects of OBT-A on sleep quality in patients with chronic migraine using the Pittsburgh Sleep Quality Index (PSQI), one of the most extensively validated instruments for assessing subjective sleep quality and sleep disturbances in clinical populations [12]. By employing a standardized and clinically meaningful measure, this study sought to provide real-world evidence on the multidimensional benefits of OBT-A and to explore the relationship between sleep improvement and migraine outcomes.

## 2. Results

**Figure 1:**
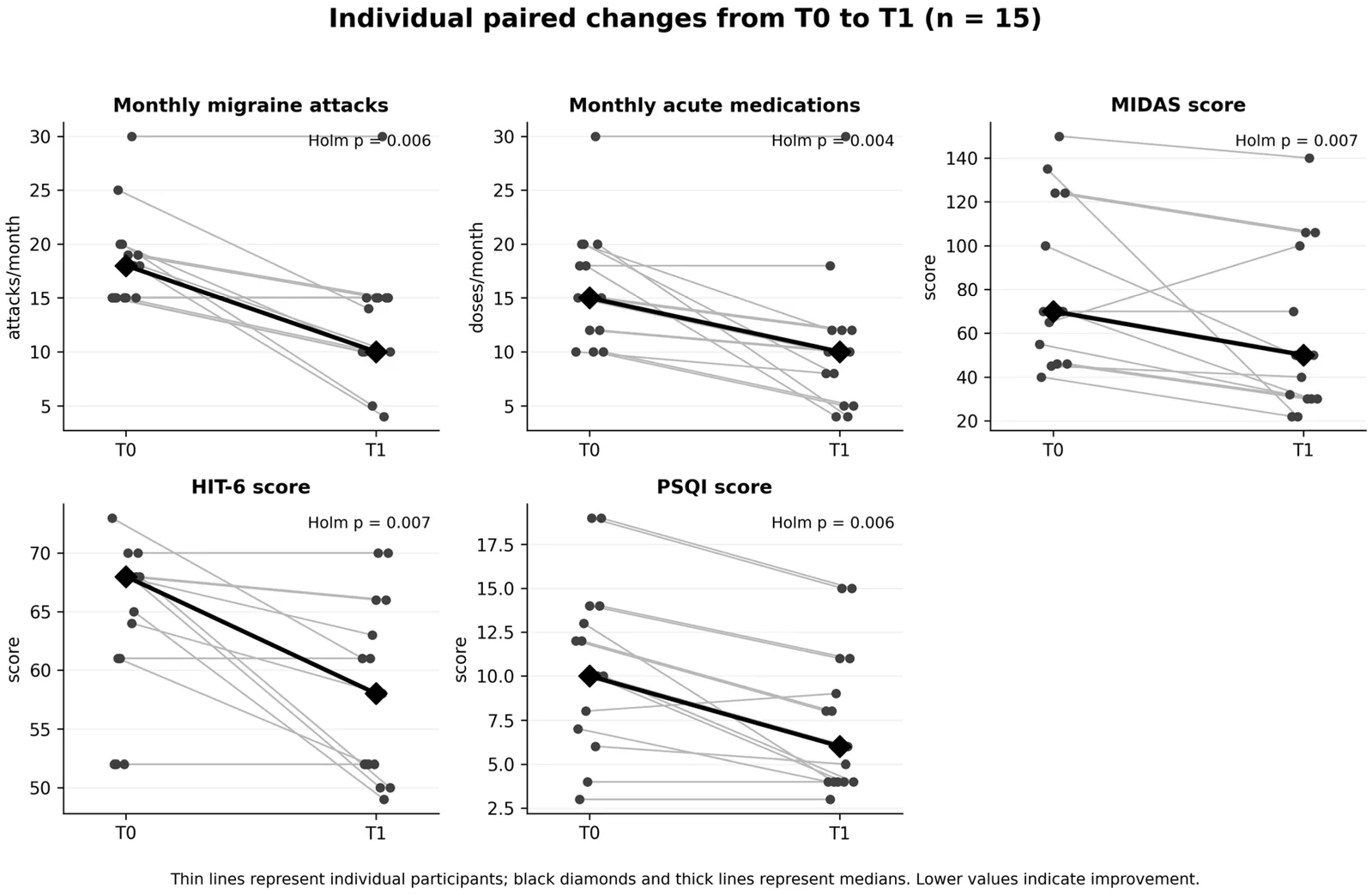
Individual paired trajectories. Individual changes from T0 to T1 for the five continuous outcomes. Gray lines represent individual participants; black diamonds and thick lines indicate median values. Lower values indicate improvement. Holm-adjusted *p* values are displayed in each panel.

Baseline characteristics are summarized in Table 1. Results of the paired analyses are presented in Table 2 and Figure 1.

Migraine frequency decreased significantly following OBT-A treatment. Median monthly migraine days (MMDs) decreased from 18 [15–19.5] at baseline to 10 [10–15] at follow-up, corresponding to a median paired reduction of −5 [−9 to −2]. The Holm-adjusted *p* value was 0.006, with a rank-biserial correlation of −1.00, indicating that all non-zero paired differences were in the same direction. At the individual level, 9 of 15 patients (60.0%) decreased from ≥15 MMDs at baseline to <15 MMDs at T1, whereas 6 patients (40.0%) remained at or above the 15-MMD threshold.

Acute symptomatic medication use also decreased significantly following treatment. Median monthly medication intake declined from 15 [12–19] to 10 [6.5–12], with a median paired change of −5 [−6.5 to −2]. The change remained significant after Holm correction (adjusted *p* = 0.004; rank-biserial correlation = −1.00).

Migraine-related disability, assessed using MIDAS, also improved significantly. Median scores decreased from 70 [50.5–112] to 50 [30–85], with a median change of −18 [−21.5 to −13]. The discrepancy between median and mean changes likely reflects the presence of both a marked improvement in one participant and a clinically relevant worsening in another.

Similarly, headache impact measured by HIT-6 showed significant improvement. Median scores decreased from 68 [61–68] to 58 [52–64.5], with a median paired change of −2 [−10.5 to 0]. Despite the relatively modest median change, all non-zero differences were favorable, resulting in a significant adjusted *p* value (0.007) and a rank-biserial correlation of −1.00.

Importantly, sleep quality also improved significantly following treatment. Median PSQI scores decreased from 10 [7.5–13.5] at baseline to 6 [4–10] after treatment, with a median paired change of −4 [−4 to −2]. The mean change was −3.3 points (95% CI −4.8 to −1.9), corresponding to a 31.1% decrease from baseline values. Twelve participants showed a reduction in PSQI score, two remained unchanged, and one worsened.

When a ≥3-point reduction was used as a clinically meaningful responder threshold, 11 of 15 patients (73.3%) met this criterion. The adjusted *p* value was 0.006, with a rank-biserial correlation of −0.97. Furthermore, the proportion of patients with pathological sleep quality (PSQI > 5) decreased from 86.7% (13/15) to 53.3% (8/15). Five participants transitioned from pathological to non-pathological sleep quality, whereas none showed the opposite transition. Although the exact McNemar test did not reach statistical significance (*p* = 0.063), likely because of the limited sample size and the small number of discordant pairs, the observed shift suggests a potential improvement in sleep quality following OBT-A treatment and warrants confirmation in larger studies.

Exploratory association analyses were performed to investigate whether improvements in sleep quality were associated with improvements in migraine-related outcomes. Changes in PSQI showed the strongest association with changes in MIDAS (Spearman ρ = 0.614, nominal *p* = 0.015), indicating that larger reductions in PSQI tended to occur in patients experiencing greater reductions in migraine-related disability. However, this association did not remain statistically significant after correction for the four exploratory comparisons (Holm-adjusted *p* = 0.060). Associations between changes in PSQI and MMDs (ρ = 0.344, *p* = 0.209), monthly acute medication use (ρ = 0.312, *p* = 0.258), and HIT-6 (ρ = 0.346, *p* = 0.206) were weaker and did not reach statistical significance; the corresponding Holm-adjusted *p* value was 0.618 for each comparison. Because changes were calculated as T1−T0 and lower values indicated improvement across all outcomes, positive correlations reflected concordant clinical improvement. Among the migraine-related outcomes, the largest correlation coefficient was observed between ΔPSQI and ΔMIDAS, whereas associations with ΔMMDs and ΔHIT-6 were weaker, indicating that the exploratory signal was predominantly related to changes in migraine-related disability.

Given the limited sample size, these findings should be regarded as exploratory and hypothesis-generating. Nevertheless, the observed trend toward improved sleep quality warrants confirmation in larger prospective studies.

## 3. Discussion

This prospective real-world observational pilot study investigated the effects of OBT-A on sleep quality in a cohort of patients with chronic migraine. Given the limited sample size and the relatively short follow-up after a single treatment cycle, the findings should be interpreted as preliminary. Nevertheless, they provide novel insights into the relationship between OBT-A treatment, sleep quality, and migraine burden and support further investigation of sleep as a clinically relevant outcome in chronic migraine management. Furthermore, the results offer an additional rationale for exploring the biological links between CGRP signaling, central sensitization, and sleep regulation. The majority of patients experienced improved subjective sleep quality following the first OBT-A treatment cycle, as reflected by the significant reduction in PSQI scores. This observation is particularly noteworthy given the high prevalence of sleep disturbances among individuals with migraine, affecting up to 79% of patients and reaching clinically significant severity in approximately 40% of cases [13,14]. Increasing evidence indicates that the relationship between migraine and sleep is bidirectional, with sleep disturbances contributing to migraine chronification, greater disability, and reduced quality of life, while recurrent migraine attacks further disrupt sleep quality and sleep architecture [15,16]. Within this framework, the observed improvement in sleep quality represents a potentially meaningful clinical benefit. However, because sleep quality improved alongside migraine frequency, disability, headache impact, and medication use, the present data do not allow determination of whether this finding reflects a direct effect on sleep-related mechanisms or occurs secondary to improvements in migraine burden. Therefore, sleep improvement should be interpreted as an associated clinical outcome rather than evidence of a specific sleep-targeted effect of OBT-A.

Following the first OBT-A treatment cycle, patients reported a median PSQI score of 6. Although this value remained within the pathological range, it corresponded to a median reduction of 4 points from baseline. Importantly, improvement in sleep quality was observed in most participants, with 12 of 15 patients showing lower PSQI scores after treatment. Although a substantial proportion of patients continued to exhibit pathological sleep quality after three months, the reduction in the prevalence of PSQI > 5 from 86.7% to 53.3% suggests a trend toward better sleep quality. However, this categorical change did not reach statistical significance (exact *p* = 0.063) and should therefore be interpreted with caution. This observation is particularly relevant considering the high migraine burden and the multiple previous preventive treatment failures characterizing our study population. Taken together, these findings suggest a clinically meaningful improvement in subjective sleep quality from a patient-centered perspective, although the clinical significance of categorical changes in sleep-status classification remains uncertain.

The role of prophylactic treatments in the relationship between migraine and sleep is currently being assessed: monoclonal antibodies targeting the calcitonin gene-related peptide (CGRP) pathway appear to have a positive effect on both migraine severity and sleep quality [17,18].

Standard preventive therapies, including antidepressants and calcium-channel blockers, may contribute to improvements in sleep quality, although their effects on sleep have been less extensively investigated than those of CGRP-targeted therapies [17]. Very few studies have examined the impact of OBT-A on sleep quality, and the results have been inconsistent. In the COMPEL study, Blumenfeld et al. assessed over 370 patients treated with OBT-A. They demonstrated that OBT-A led to a significant reduction in migraine severity and a reduction in PSQI scores [19]. Furthermore, only 40.9% recorded a reduction of ≥3 points in their PSQI scores at the end of the study. In contrast, other studies have not observed any significant improvement in sleep quality following OBT-A therapy [13].

Sleep quality appears to represent an integral component of the chronic migraine phenotype rather than a simple comorbidity. The parallel improvements observed across migraine frequency, acute medication use, disability, headache impact, and sleep quality suggest that these domains are closely interconnected and may be influenced by partially overlapping biological mechanisms. Although causality cannot be inferred from the present observational study, the findings support the concept that sleep quality may be both a consequence and a determinant of migraine burden.

Sleep disturbances and migraine are closely interconnected, although the underlying mechanisms remain only partially understood. Experimental and clinical evidence suggests that impaired sleep may contribute to migraine chronification, while recurrent migraine attacks may further disrupt sleep quality and sleep architecture [20,21]. Reduced REM sleep and alterations in REM-to-wake transition mechanisms have been described in migraine, and REM sleep deprivation has been associated with increased migraine susceptibility and worsening of allodynia [21,22]. Migraine worsening is also frequently associated with sleep disorders such as insomnia, obstructive sleep apnea, parasomnias, and sleep-related movement disorders, potentially through reduced pain thresholds and increased cortical excitability [23,24,25]. Previous studies have further suggested that the hypothalamus may represent an important anatomical substrate linking migraine pathophysiology and sleep regulation, with neurotransmitter systems such as serotonin and orexin potentially contributing to this interaction [20,23,26].

The biological mechanisms underlying the association between OBT-A treatment and improved sleep quality remain uncertain. OBT-A inhibits the release of CGRP and other pronociceptive neuropeptides and may reduce peripheral and central sensitization, mechanisms that contribute to migraine improvement [8,10,11,27]. Because all major migraine-related outcomes showed improvement during follow-up, it is plausible that the observed improvements in sleep quality were partly driven by reductions in headache burden and its consequences. Although CGRP signaling and central sensitization have been implicated in both migraine and sleep regulation [3,4], the present observational study was not designed to determine whether OBT-A exerts an independent effect on sleep physiology. Therefore, the present findings should be interpreted as evidence of an association rather than evidence of a direct sleep-specific effect of OBT-A. Future controlled studies incorporating objective sleep measures and mediation analyses will be necessary to clarify these relationships.

Our findings also support the efficacy of OBT-A in the treatment of CM. Previous studies had already demonstrated the efficacy of OBT-A therapy in CM and the persistence of the effect [28]. Our patient sample is, of course, limited, but even after the first OBT-A treatment cycle, patients showed a significant improvement in migraine severity.

In particular, the median frequency of MMDs decreased from 18 to 10, reflecting a substantial reduction in migraine burden after the first OBT-A treatment cycle. In addition to the reduction in median monthly migraine days from 18 to 10, 9 of 15 patients (60%) no longer fulfilled diagnostic criteria for chronic migraine at follow-up. This represents a significant benefit for patients, as CM is associated with excessive medication use, substantial disability, and impaired quality of life [15,29]. The migraine burden, quantified by the MIDAS and HIT-6 scores, was also found to have improved significantly, highlighting a close correlation between sleep and quality of life.

Moreover, this exploratory association analysis provides additional insight into these parallel changes. Improvement in PSQI was most strongly associated with improvement in MIDAS (Spearman ρ = 0.614), whereas associations with migraine frequency, acute medication use, and HIT-6 were weaker. Although the PSQI–MIDAS association reached nominal statistical significance, it did not remain significant after correction for multiple exploratory comparisons. This finding should therefore be considered hypothesis-generating and may indicate that improvement in subjective sleep quality is more closely related to changes in the broader disability associated with migraine than to headache frequency alone. However, the present analysis cannot establish directionality or causality, and confirmation in larger longitudinal cohorts incorporating objective sleep measures and mediation analyses is required.

Taken together, these findings indicate that improvements in sleep quality occurred in parallel with improvements in multiple dimensions of migraine-related burden. However, the present study does not allow conclusions regarding whether sleep improvement represents an effect independent of headache reduction.

From a clinical perspective, improvements in sleep quality may represent a meaningful therapeutic benefit extending beyond headache control. This aspect may be particularly relevant in CM, where disability, medication overuse, and impaired quality of life are major determinants of overall disease burden [29]. Viewed within the context of contemporary models of migraine pathophysiology, the parallel improvements observed across sleep quality, headache frequency, disability, and medication use suggest that these domains are closely interconnected. However, the present study cannot determine the directionality of these relationships or the extent to which sleep improvement is independent of improvements in migraine severity.

Several limitations should nevertheless be acknowledged. First, the sample size was small, comprising only 15 patients. However, as previously noted, this analysis represents a preliminary evaluation of an ongoing prospective study and should therefore be considered hypothesis-generating rather than definitive. In addition, patients were evaluated after only one treatment cycle and a relatively short follow-up period. Longer follow-up periods will be necessary to determine the persistence of the observed effects on both migraine severity and sleep quality. Second, the observational design and the absence of a control group preclude any causal inference regarding the relationship between OBT-A administration and improvement in sleep quality. Consequently, the study cannot determine whether the improvements in sleep quality reflect a direct effect of OBT-A on sleep-related pathways or are secondary to concurrent improvements in migraine frequency, disability, headache impact, and medication use. It therefore remains uncertain whether the observed sleep benefits are directly mediated by biological effects of OBT-A on pain-processing and sleep-regulating pathways or whether they occur indirectly because of reduced headache burden.

Third, sleep quality was assessed exclusively using the PSQI, a validated and widely used patient-reported outcome measure. Although this approach provides clinically meaningful information regarding patients’ subjective perception of sleep, objective measurements such as actigraphy or polysomnography were not available. Consequently, potential treatment-related modifications in sleep architecture, REM sleep regulation, sleep efficiency, or nocturnal awakenings could not be investigated. Furthermore, despite the requirement for stable concomitant therapies throughout the observation period, the possible influence of preventive medications, psychiatric comorbidities, and other sleep-related conditions cannot be completely excluded. The limited sample size prevented meaningful subgroup analyses capable of addressing these potentially relevant confounders. Unmeasured sleep-related factors may have influenced the observed results and should be systematically assessed in future studies. Finally, all participants were recruited from a tertiary headache center and were characterized by a high disease burden and multiple previous preventive treatment failures. Although this enhances the clinical relevance of the findings in a real-world chronic migraine population, caution is warranted when extrapolating the results to patients with less severe disease.

Despite these limitations, the study also has important strengths. To our knowledge, this is one of the first prospective real-world observational pilot studies specifically designed to investigate changes in sleep quality following OBT-A treatment in CM using a validated sleep assessment instrument. Furthermore, it evaluates sleep quality as a clinically relevant outcome alongside migraine frequency, disability, headache impact, and medication use, thereby providing a more comprehensive assessment of treatment benefit. This study’s strengths also include its prospective design, the use of validated instruments to assess both migraine-related outcomes and sleep quality, and the real-world evaluation of patients receiving OBT-A in routine clinical practice. The consistency of improvement observed across migraine frequency, medication use, disability, headache impact, and sleep quality strengthens the clinical relevance of the findings and supports the hypothesis that these domains are closely interconnected.

In conclusion, this prospective real-world observational pilot study provides preliminary evidence that treatment with OBT-A is associated with improved subjective sleep quality in patients with CM, occurring alongside reductions in headache frequency, disability, headache impact, and medication use. Because of the uncontrolled observational design, no conclusions can be drawn regarding whether sleep improvement represents a direct effect of OBT-A or occurs secondary to improvement in migraine-related outcomes. These findings nevertheless highlight sleep quality as an important dimension of migraine burden and support further controlled studies aimed at clarifying the mechanisms underlying the observed association.

## 4. Materials and Methods

This prospective real-world observational pilot study was conducted at a tertiary headache center, between 1 January and 31 December 2025. All consecutive patients diagnosed with chronic migraine (CM) according to the International Classification of Headache Disorders, 3rd edition (ICHD-3) criteria [30] were screened for inclusion. Patients were enrolled if they were initiating treatment with OBT-A for the first time during the study period. No patients with MOH were included in the study.

OBT-A was administered according to the PREEMPT protocol, with a standard starting dose of 155 U and the possibility of dose escalation up to 195 U using the “follow-the-pain” strategy, according to clinical judgment and current recommendations derived from PREEMPT and subsequent real-world studies [31]. All patients received the standard 155 U dose during the first OBT-A treatment cycle evaluated in this study. Dose escalation up to 195 U, when clinically indicated according to current recommendations, was considered only at the T1 visit following assessment of treatment response and therefore could not have influenced the outcomes reported for the first treatment cycle analyzed in the present study [31,32].

At baseline (T0), all participants underwent a comprehensive neurological evaluation and completed a standardized assessment battery including:

- Migraine Disability Assessment (MIDAS);
- Headache Impact Test-6 (HIT-6);
- Pittsburgh Sleep Quality Index (PSQI).

These instruments were used to evaluate migraine-related disability, headache impact, and sleep quality, respectively.

Patients were allowed to continue stable preventive and acute migraine treatments during the observation period, provided that no major treatment modifications occurred between T0 and T1. Moreover, we did not deeply investigate psychiatric comorbidities, but we excluded patients receiving treatment with benzodiazepines or neuroleptics.

Patients with diagnosed sleep disorder (narcolepsy, REM sleep behavior disorders) or in stable treatment with hypnotic drugs were excluded from the study. We did not perform instrumental assessments such as actigraphy or polysomnography; consequently, potential treatment-related changes in sleep architecture, REM sleep regulation, sleep efficiency, or nocturnal awakenings could not be investigated. In this context, the possible influence of preventive medications, psychiatric comorbidities, and other sleep-related conditions cannot be completely excluded.

Follow-up assessments (T1) were performed three months after the first OBT-A treatment cycle and immediately before the second scheduled OBT-A administration. At T1, all clinical assessments and questionnaires were repeated to evaluate changes in migraine burden and sleep quality.

All participants provided written informed consent prior to enrolment. The study was conducted in accordance with the principles of the Declaration of Helsinki and complied with all applicable institutional and national regulations.

### Statistical Analysis

Eighteen patients met the eligibility criteria and were initially considered for enrolment. Two patients declined participation, and one discontinued treatment before the second injection cycle for personal reasons. Consequently, the final study population consisted of 15 patients. Among them, four patients continued stable preventive migraine treatments during the observation period (two with calcium-channel blockers, one with a tricyclic antidepressant and one with a CGRP-targeting therapy—galcanezumab).

The analysis included all participants with paired baseline (T0) and follow-up (T1) data for the five prespecified outcomes:

1. Monthly migraine days (MMDs);
2. Monthly use of acute symptomatic medications;
3. Migraine Disability Assessment (MIDAS);
4. Headache Impact Test-6 (HIT-6);
5. Pittsburgh Sleep Quality Index (PSQI).

For all outcomes, lower values were considered indicative of clinical improvement. The dichotomous classification of pathological sleep quality (PSQI > 5) was analyzed separately.

The study population had a mean age of 49.7 years (standard deviation [SD] 12.1 years; median age 51 years), and 13 of 15 participants (86.7%) were female. Outcome data were complete for all patients included.

Given the small sample size and non-normal distribution observed for several variables, particularly MIDAS scores, continuous variables are presented as median and interquartile range (IQR). Absolute change was calculated as T1 minus T0; therefore, negative values indicate clinical improvement.

The Wilcoxon signed-rank test was selected as the primary inferential method and applied to all five continuous outcomes. To account for multiple comparisons, family-wise error was controlled using the Holm correction.

Effect sizes were estimated using the matched-pairs rank-biserial correlation, with negative values indicating a shift toward lower scores at follow-up. Mean changes and corresponding 95% confidence intervals (95% CIs) were also calculated as descriptive measures of the magnitude of treatment effect. The matched-pairs rank-biserial correlation ranges from −1 to +1 and reflects the predominant direction of paired changes; values approaching −1 indicate that non-zero changes are consistently toward lower follow-up values, whereas values approaching +1 indicate changes toward higher values. For rank-biserial effect-size estimation, zero paired differences were excluded before ranking and did not contribute to either the positive or negative rank sum. Mean paired changes and corresponding 95% confidence intervals were additionally reported as supplementary descriptive measures of change magnitude and were not used for inferential purposes. Given the small sample size and non-normal distribution of several outcomes, median-based measures remained the primary descriptive statistics. Percentage changes were also reported solely as supplementary descriptive measures to facilitate interpretation of the magnitude of change relative to baseline values.

Individual patient responses were categorized as improved, unchanged, or worsened according to the direction of the paired difference. Among patients presenting with pathological sleep quality at baseline (PSQI > 5), changes in classification status were evaluated using the exact McNemar test.

Given the exploratory nature of this pilot study, no formal sample-size calculation was performed. No imputation procedures were required, as outcome data were complete for all analyzed participants.

To explore whether improvements in sleep quality were associated with migraine-related outcomes, exploratory Spearman rank correlations were calculated between the absolute change in PSQI score and the corresponding changes in MMDs, monthly acute medication use, MIDAS, and HIT-6. Changes were calculated as T1 minus T0; therefore, negative values indicated clinical improvement, and positive correlation coefficients reflected concordant changes across outcomes. Rank-based correlations were selected because of the limited sample size, non-normal distributions, and the presence of tied observations. Given the hypothesis-generating nature of these analyses, both nominal and Holm-adjusted *p* values for the four correlations were reported. These analyses were exploratory and were not intended to provide confirmatory evidence regarding mediation or causality.

Statistical analyses were performed using Jamovi version 2.6.44 (The Jamovi Project, Sydney, Australia). ChatGPT (OpenAI; GPT-5.6 Sol) was used as a generative artificial intelligence (GenAI)-assisted tool to support the formulation and execution of selected exploratory statistical analyses, to generate Figure 1, and to assist in interpreting the outputs. All statistical procedures, numerical results, and interpretations generated with GenAI assistance were independently checked and validated by the authors against the original data and the Jamovi output. The authors take full responsibility for the accuracy and integrity of the reported analyses.

All statistical tests were two-tailed, and a *p* value < 0.05 was considered statistically significant.

## Figures and Tables

**Table 1:** Baseline characteristics of the sample.

Demographic Characteristics	
Age, years	49.7 ± 12.1
**Sex, n (%)**	
Female	13 (86.7)
Male	2 (13.3)
**Clinical history and prior treatment**	
Migraine duration, years	12 (8–20)
Duration of chronic migraine, years	2 (2–5)
Number of previous preventive treatments	4 (4–6)
Any recorded comorbidity, n (%)	6 (40.0)
**Acute medication classes, n (%)**	
Triptans	12 (80.0)
Non-steroidal anti-inflammatory drugs	12 (80.0)
Paracetamol	2 (13.3)
Opioids	0 (0.0)
**Previous preventive treatment classes, n (%)**	
Beta-blockers	9 (60.0)
Calcium-channel blockers *	10 (66.7)
Antiepileptic drugs	10 (66.7)
Tricyclic antidepressants *	10 (66.7)
Selective serotonin reuptake inhibitors	10 (66.7)
Other treatments	8 (53.3)
CGRP-targeting therapies *	9 (60.0)
**Baseline migraine burden and sleep quality**	
MMDs	18 (15–19.5)
Monthly acute medication use	15 (12–19)
MIDAS score	70 (50.5–112)
HIT-6 score	68 (61–68)
PSQI score	10 (7.5–13.5)
Pathological PSQI, n (%)	13 (86.7)

Data are presented as mean ± standard deviation, median [interquartile range], or n (%), as appropriate. Percentages are based on N = 15 unless otherwise indicated. Acute and preventive medication classes are non-mutually exclusive. * = stable preventive migraine treatments continued after T0 (two calcium-channel blockers, one tricyclic antidepressant and one CGRP-targeting therapy—galcanezumab). **Abbreviations:** CGRP, calcitonin gene-related peptide; MMDs, monthly migraine days; MIDAS, Migraine Disability Assessment; HIT-6, Headache Impact Test-6; PSQI, Pittsburgh Sleep Quality Index.

**Table 2:** Paired Changes from T0 to T1.

Outcome	T0 Median [IQR]	T1 Median [IQR]	Median Δ [IQR]	Adjusted *p*/Effect	Individual Response
MMDs	18 (15–19.5)	10 (10–15)	−5 (−9–−2)	*p* = 0.006; r = −1.00	11 improved; 4 unchanged; 0 worsened
Monthly acute medications	15 (12–19)	10 (6.5–12)	−5 (−6.5–−2)	*p* = 0.004; r = −1.00	13 improved; 2 unchanged; 0 worsened
MIDAS score	70 (50.5–112)	50 (30–85)	−18 (−21.5–−13)	*p* = 0.007; r = −0.79	13 improved; 1 unchanged; 1 worsened
HIT-6 score	68 (61–68)	58 (52–64.5)	−2 (−10.5–0)	*p* = 0.007; r = −1.00	9 improved; 6 unchanged; 0 worsened
PSQI score	10 (7.5–13.5)	6 (4–10)	−4 (−4–−2)	*p* = 0.006; r = −0.97	11 with ≥3-point reduction; 1 with 1-point reduction; 2 unchanged; 1 worsened
PSQI pathological, n (%)	13 (86.7)	8 (53.3)	−5 cases	exact *p* = 0.063	5 reverted; 0 developed

Δ = T1 − T0; negative values indicate improvement. Continuous outcomes were tested using two-sided Wilcoxon signed-rank tests with the Pratt method for zero differences; *p* values were Holm-adjusted across the five outcomes. r = matched-pairs rank-biserial correlation. For the matched-pairs rank-biserial correlation, values approaching −1 indicate increasingly consistent changes toward lower follow-up values; r = −1.00 indicates that all non-zero paired differences occurred in this direction; *p* values were Holm-adjusted across five outcomes. The binary PSQI comparison used an exact paired binomial/McNemar test and was not included in the Holm family. **Abbreviations:** MMDs, monthly migraine days; IQR, interquartile range; MIDAS, Migraine Disability Assessment; HIT-6, Headache Impact Test-6; PSQI, Pittsburgh Sleep Quality Index; Δ, change from T0 to T1; r, matched-pairs rank-biserial correlation.

## Data Availability

The original contributions presented in this study are included in the article. Further inquiries can be directed to the corresponding author.
